# Implementation of foot thermometry plus mHealth to prevent diabetic foot ulcers: study protocol for a randomized controlled trial

**DOI:** 10.1186/s13063-016-1333-1

**Published:** 2016-04-19

**Authors:** Maria Lazo-Porras, Antonio Bernabe-Ortiz, Katherine A. Sacksteder, Robert H. Gilman, German Malaga, David G. Armstrong, J. Jaime Miranda

**Affiliations:** CRONICAS Center of Excellence in Chronic Diseases, Universidad Peruana Cayetano Heredia, Av. Armendáriz 497, Miraflores, Lima 18, Peru; School of Public Health and Administration, Universidad Peruana Cayetano Heredia, Av. Honorio Delgado 430, Urb. Ingeniería, Lima 31, Peru; Department of International Health, Johns Hopkins Bloomberg School of Public Health, Johns Hopkins University, 615 N Wolfe Street, Baltimore, MD USA; Área de Investigación y Desarrollo, Asociación Benéfica PRISMA, Carlos Gonzales 251, Maranga, Lima 32, Peru; Department of Medicine, School of Medicine, Universidad Peruana Cayetano Heredia, Av. Honorio Delgado 430, Urb. Ingeniería, Lima 31, Peru; Southern Arizona Limb Salvage Alliance (SALSA), Department of Surgery, University of Arizona College of Medicine, Tucson, AZ USA

**Keywords:** Diabetic neuropathies, Thermometry, Diabetes mellitus, Type 2 ulcer, mHealth

## Abstract

**Background:**

Diabetic foot neuropathy (DFN) is one of the most important complications of diabetes mellitus; its early diagnosis and intervention can prevent foot ulcers and the need for amputation. Thermometry, measuring the temperature of the feet, is a promising emerging modality for diabetic foot ulcer prevention. However, patient compliance with at-home monitoring is concerning. Delivering messages to remind patients to perform thermometry and foot care might be helpful to guarantee regular foot monitoring. This trial was designed to compare the incidence of diabetic foot ulcers (DFUs) between participants who receive thermometry alone and those who receive thermometry as well as mHealth (SMS and voice messaging) over a year-long study period.

**Methods/design:**

This is an evaluator-blinded, randomized, 12-month trial. Individuals with a diagnosis of type 2 diabetes mellitus, aged between 18–80 years, having a present dorsalis pedis pulse in both feet, are in risk group 2 or 3 using the diabetic foot risk classification system (as specified by the International Working Group on the Diabetic Foot), have an operating cell phone or a caregiver with an operating cell phone, and have the ability to provide informed consent will be eligible to participate in the study. Recruitment will be performed in diabetes outpatient clinics at two Ministry of Health tertiary hospitals in Lima, Peru.

Interventions: participants in both groups will receive education about foot care at the beginning of the study and they will be provided with a thermometry device (TempStat™). TempStat™ is a tool that captures a thermal image of the feet, which, depending on the temperature of the feet, shows different colors. In this study, if a participant notes a single yellow image or variance between one foot and the contralateral foot, they will be prompted to notify a nurse to evaluate their activity within the previous 2 weeks and make appropriate recommendations. In addition to thermometry, participants in the intervention arm will receive an mHealth component in the form of SMS and voice messages as reminders to use the thermometry device, and instructions to promote foot care.

Outcomes: the primary outcome is foot ulceration, evaluated by a trained nurse, occurring at any point during the study.

**Discussion:**

This study has two principal contributions towards the prevention of DFU. First, the introduction of messages to promote self-management of diabetes foot care as well as using reminders as a strategy to improve adherence to daily home-based measurements. Secondly, the implementation of a thermometry-based strategy complemented by SMS and voice messages in an LMIC setting, with wider implications for scalability.

**Trial registration:**

This study is registered in ClinicalTrials.gov: Identifier NCT02373592.

**Electronic supplementary material:**

The online version of this article (doi:10.1186/s13063-016-1333-1) contains supplementary material, which is available to authorized users.

## Background

There are an estimated 392 million people worldwide living with type 2 diabetes mellitus (T2DM), 80 % of whom live in low- and middle-income countries (LMICs) [1]. Diabetic foot neuropathy (DFN) is a frequent complication and has enormous impacts on patients, families, and society. According to information from the US, 60–70 % of people with diabetes will develop peripheral neuropathy [2] and, of those, up to 25 % will develop a foot ulcer (DFU), a significant prognostic factor for future foot amputation [3].

While data from Peru is limited, our preliminary study found a 57 % prevalence of DFN at a National Public Hospital, 20 % of whom are considered to be high-risk for developing a DFU due to the presence of deformities [4]. In addition, the economic burden associated with DFUs – a mostly preventable condition – is enormous. Some projections indicate that the cost of treating one case of DFU with surgical debridement ranged between US$1,022 and US$1,404 in Peru, which is 200 % of the average monthly family income in Lima, Peru [5]. To avoid these preventative and costly complications, necessary strategies including patient education, foot care, and the promotion of appropriate footwear are needed.

Tools to identify early signs of foot damage or inflammation have the potential to reduce the incidence of foot ulceration and amputation. Thermometry, for example, measures superficial skin warmth and is a promising emerging modality to evaluate and manage early signs and help to prevent DFUs. In three independent clinical trials, Lavery and Armstrong found that utilization of thermometry among individuals with diabetes at high-risk of developing a DFU reduced rates of recurring ulcers four- to ten-fold [6–8]. Moreover, a systematic review indicated that the use of temperature monitoring is an effective way to predict and prevent diabetic foot ulceration [9].

Recently, a novel self-assessment device, TempStat™ (Visual Footcare Technologies, LLC, South Salem, NY, USA), a plastic panel with two polycarbonate plastic pads, has become available. The plastic pads are constructed of liquid crystalline cholesteric esters that react to skin surface temperature and change color to reflect temperature [10]. Frykberg et al*.* showed that TempStat™ can detect “alarm signs,” represented by a yellow color change, and the results positively correlate to temperature findings of infrared thermometer, the “gold standard” of thermometry devices [11]. As such, the use of TempStat™ is a low-cost, at-home, and patient-friendly way to prevent the development of DFUs and other complications such as amputation. The caveat for any thermometry device, however, is that the patient is required to adhere to self-assessment to achieve the observed efficacy; i.e., published results suggest that participants needed to evaluate their foot temperature on at least 50 % of the days to reduce the risk of foot ulceration [7].

In Peru and many other LMICs, clinical consultations are very short, lasting on average 10 − 15 minutes, which minimizes the time allowed for patient education [12, 13]. In addition, there are significant barriers in physician-patient communication [14], and medical treatment adherence is complicated by poor patient literacy and low socioeconomic status [15, 16]. The lack of resources for specialized diabetic follow-up programs leaves patients without knowledge of their disease process and unable to engage in self-care decision-making. Because of these problems, we need to identify ways to facilitate improved patient education and self-management for prevention of diabetic complications.

mHealth – the use of mobile technology to promote wellness – can close the gap between patient behaviors and the healthcare system. The use of Short Message Service (SMS) for diabetes management was evaluated by two systematic reviews, noting that patients with T2DM who received text message interventions showed improvements in Self-Efficacy for Diabetes and Diabetes Social Support Interview scores [17], and thus could improve clinically diabetes-related health outcomes [18].

Thermometry and SMS have independently demonstrated enormous potential to lead to positive diabetes-related outcomes in high-income countries. To strengthen these approaches in a LMIC in South America, we propose to use both modalities to evaluate the efficacy of a combination of foot thermometry with mHealth reminders, using SMS and voice messaging, in reducing the risk of foot ulceration in Peru.

## Methods/design

### Objectives

The main objective of this study is to compare the 1-year incidence of DFU in two arms: the intervention arm, receiving thermometry and mHealth reminders; and the control arm receiving thermometry alone. Our hypothesis is that subjects receiving SMS and voice messaging reminders will have a lower incidence of DFU than subjects who do not receive reminders. Additionally, we will compare patient compliance with thermometer use in both arms with a temperature-recording logbook.

#### Primary specific aim

1. Compare the incidence of DFU during the study period between those who receive thermometry alone and those who receive thermometry plus SMS and voice messaging

#### Secondary specific aims

1. Compare the compliance of foot thermometer use between the two study arms
2. Compare the frequency of alarm signs reported to the study nurse between the two study arms
3. Compare the frequency of alarm signs reported in the patient’s temperature-recording logbooks between the two study arms
4. Compare the incidence of DFU according to pre-specified sub-groups: caregiving status, use of insoles and/or orthopedic shoes
5. In the intervention-only arm, compare the incidence of DFU by recipient of the messaging intervention (patient versus caregivers)

### Study design

This is a physician- and evaluator-blinded, 1-year, randomized clinical trial with two parallel groups, and a 1:1 allocation.

### Participant recruitment and selection criteria

**Fig. 1:**
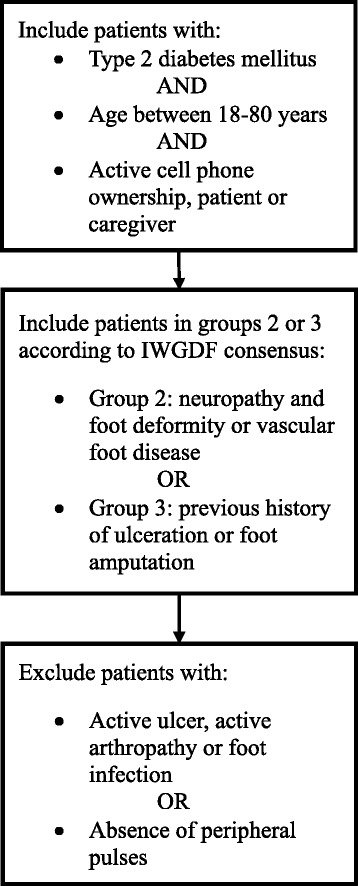
Process of screening evaluation. Legend: description of the inclusion and exclusion criteria of the study and screening process. IWGDF: International Working Group on the Diabetic Foot

Recruitment will be performed in the outpatient clinics of the aforementioned hospitals. Subjects will be eligible if they have a diagnosis of T2DM, are between 18 and 80 years of age, are in risk group 2 or 3 using the diabetic foot risk classification system (as specified by the International Working Group on the Diabetic Foot) [19–21], have a present dorsalis pedis pulse in both feet, have an operating cell phone or a caregiver with an operating cell phone, and have the ability to provide informed consent. Subjects will not be eligible for enrollment if they have current foot ulcers, active Charcot osteoarthropathy, severe peripheral arterial disease, or foot infection (Fig. 1).

Foot evaluation to place subjects into an eligible risk group will be performed using the diabetic foot risk classification system [22] as shown in Fig. 1. This evaluation includes a short questionnaire about previous history of ulceration and/or partial foot amputation, foot evaluation to detect deformities such as hallux valgus, rigid toe contractures (such as hammer or claw toes), and prominent metatarsal heads [19], and neuropathy testing using the vibration perception threshold and the Semmes-Weinstein monofilament (SWF) [22].

### Baseline data collection

**Fig. 2:**
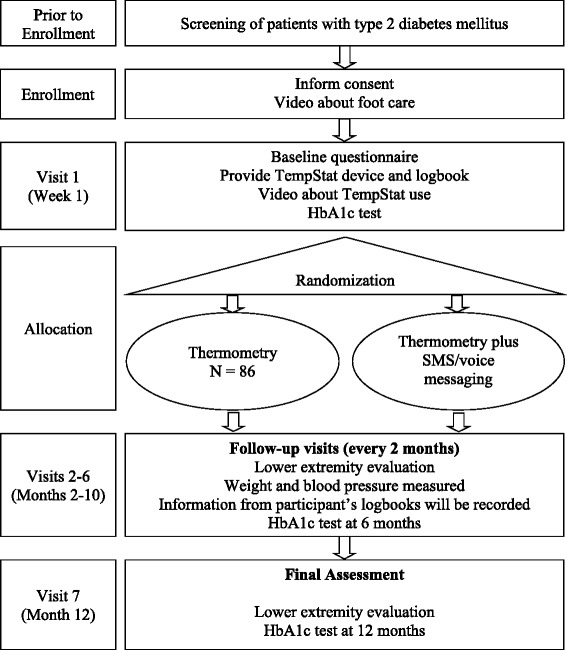
Flow chart. Legend: description of the trial. HbA1c: Glycated Hemoglobin SMS: Short Message Service

We will record participants’ baseline data through questionnaires during visit 1, 1 week after enrollment (see Fig. 2). This information will include: (1) demographic evaluation, (2) socioeconomic evaluation, (3) lifestyles including tobacco, physical activity, and alcohol consumption, (4) mental health, specifically depression, (5) participants’ history of cardiovascular disease and diabetes, (6) current pharmacological treatment for diabetes, (7) pattern of use of insoles and orthopedic shoes, (8) mobile phone literacy, (9) anthropometric evaluation, and (10) blood pressure evaluation.

Blood samples, to determine baseline levels of glycated hemoglobin, will be conducted at baseline, and at 6-month and at 12-month follow-up visits.

### Randomization

We will follow the Consolidated Standards of Reporting Trials (CONSORT) Statement of recommendations for the reporting of randomized trials [23]. Stratification and blocking randomization, using hospital site as a single strata, will be performed to generate a random allocation sequence. Stratification by recruiting hospital site will be used to ensure that the numbers of participants are closely balanced within intervention arms.

Subjects will be randomized to one of the two intervention arms via opaque, sealed envelopes, each bearing on the outside only the name of the hospital and a code. The preparation of envelopes will be perform using an independent researcher. Physicians providing care to study participants in recruiting sites will be blinded to the study arm allocation, and randomization will occur between 1 and 6 days after baseline data collection.

Study nurses will assign study codes to each of the study participants and, independently, codes will have been randomly assigned into the intervention arm or the control group. The study coordinator will be responsible for opening the sealed envelopes and informing participants about their assigned study arm as per the random list, as well as maintaining concealment. The subjects will be instructed not to discuss their treatment group assignment with the blinded evaluator.

### Intervention

As with any other complex intervention, we have used the recently proposed TIDieR –template for intervention description and replication checklist and guide – recommendations to describe our intervention [24]. Briefly, all study participants will receive education about foot care at the beginning of the study and they will all be provided with a thermometry device (TempStat™, Visual Footcare Technologies, LLC, South Salem, NY, USA). Participants in the intervention arm will receive, in addition to thermometry, an mHealth component as reminders in the form of SMS and voice messages.

#### Physical and informational materials

Videos were the chosen mechanism to standardize the education about foot care at the beginning of the study, and also to standardize the information about use of the TempStat™. In order to develop the content of our information materials, a communicator initially prepared a questionnaire to assess preferences about education on healthy habits through audiovisual media and to assess preferences related to video format. This was accomplished through a small pilot study conducted in diabetes outpatient clinics, with 30 volunteers, over a 2-week-long period. In parallel, a literature search was conducted to gather information to explain the etiology and risk factors for the development of neuropathy and ulcers, as well as to obtain recommendations for foot care practices and early signs of ulceration. After these procedures, three guides were prepared, two focused on foot care and one guide was oriented to provide instructions on the use of the TempStat™ device. Each of the guides was then used to generate three videos, all of them produced by an audiovisual specialist. All videos were validated by physicians and patients with T2DM. The two videos about foot care are available at https://youtu.be/qZm2z5Wbwg0 and https://youtu.be/3o0CkEmWeXk, and the one on how to use the TempStat™ device is available at https://youtu.be/KotgANRXoIw.

In addition to the videos, all study participants will be provided with a TempStat™ foot thermometer device. They will be instructed to use the device daily and will be asked to record their measurements in a temperature-recording logbook to be provided by the study nurse.

Finally, on the mHealth intervention arm only, SMS and voice messages will be sent to participants. The design and development process of these messages are provided as Additional file 1.

#### Procedures, activities, and processes

##### Enrollment visit

After screening, informed consent will be obtained from all participants by the study’s nurse; patients will be enrolled and a video about foot care will be shown to each participant. All of these procedures occur at the same visit, also known as the pre-screening and enrollment visit. The two videos about foot care will be shown to participants during enrollment (see Fig. 2).

##### Visit 1

One week after enrollment (see Fig. 2), the TempStat™ will be provided to each participant together with a video about its use. Participants’ usage of the TempStat™ will be observed by a study nurse to verify that they use it adequately. Some alarm signs have been pre-defined on the TempStat™ and the study nurse will train participants to detect them. These include: (1) yellow spots in any area of any feet for two consecutive days, (2) different colors in contralateral areas of the feet for two consecutive days, or (3) a dermal lesion. In any of these three scenarios, subjects will be instructed to contact the study nurse by phone or SMS. For the first two types of alarm signs, the contact nurse will ask about the presence of any lesions as well as the patient’s activity in the last 2 weeks. The nurse will also provide recommendations on how to decrease activity until temperatures normalize. If alarm signs continue for more than 1 week after the telephone consultation, a face-to-face evaluation will be requested to assess the patient for infection and/or a masked injury. If a dermal lesion is present, participants will be asked to be evaluated promptly by a nurse blind to the intervention. When a DFU, the main outcome, has been confirmed, the study nurse will direct the patients to receive professional specialty care.

##### Intervention arm

In the intervention arm, which includes an mHealth component, participants will receive two reminder messages and six foot-care promotion messages during the study period. The content of these eight messages has been developed and validated through both via SMS and voice messaging (see Additional file 1). During the first 2 weeks of the intervention, daily reminders to use the TempStat™ will be sent Monday to Friday via both SMS and voice messaging. Thereafter, for the remaining 50 weeks, patients will only receive two messages per week: one SMS and one voice message with the content alternating between reminders to use the TempStat™ device and promotion of foot care.

#### Intervention provider

Each participating site will have two study nurses and one study coordinator.

Nurses will have between 1 and 5 years of previous experience in diabetes care and will receive, as part of this study, a specific 2-day training in foot care, foot evaluation – vibration perception threshold (VPT), SWF, and pulse evaluation – as well as a description of the study procedures.

The study coordinator, an early career physician, will act as study coordinator and will provide support during the recruitment process. Study coordinators will have experience in epidemiological tools and research, and will be trained in detail in all study procedures.

#### Modes of delivery of the intervention

Recruitment of participants will be face-to-face. General education about foot care and the use of the TempStat™ will be delivered by audiovisual methods as previously described. Participants, under the observation of the study nurse, will perform a sample exercise using the TempStat™. After this, participants will then be asked to repeat the measurements every day at home and to record their observations in a temperature-recording logbook.

For the mHealth component, SMS and voice messages will be delivered to the participant’s or caregiver’s cell phones through an automated software system. Every week the system will be evaluated by the study coordinator to verify its functionality. More information about the software is available in Additional file 2.

#### Type of locations

Much of the intervention is about self-management of foot care in diabetes and occurs at home. Yet, the recruitment and enrollment into the study will be conducted at two third-level hospitals located in Lima, the capital of Peru: Hospital Nacional Cayetano Heredia and Hospital Nacional Arzobispo Loayza. Each hospital recorded over 50,000 outpatient visits of patients with diabetes in 2011 [25]. Both hospitals provide care for subjects of low socioeconomic status, much of the hospital care is subsidized through a national healthcare insurance but out-of-pocket payments for laboratory services and medicines are still very significant for this population [26]. Activities related to this protocol began in late September 2015 and are expected to continue through 2017.

#### When and how much?

After enrollment, participants will visit the clinic 1 week later to receive the TempStat™ device and instructions to use it. From that point, participants will visit the clinic every 2 months until the 12-month visit as shown in Fig. 2.

For the mHealth, during the first 2 weeks into study, the intervention arm will receive daily reminders to use the TempStat™ via both SMS and voice messaging. Thereafter, for the remaining 50 weeks, patients will only receive two messages per week: one SMS and one voice message, with content alternating between reminders to use TempStat™ and promotion of foot care. All messages will be delivered at 8 a.m.

#### Tailoring

Some form of tailoring occurs at the mHealth intervention arm. All the SMS will include the name of the participant (“Dear *participant’s name*, …) on, but not in, the voice messages. A minimal element of additional tailoring is anticipated to occur with all study participants, both intervention and control arms, during their face-to-face interactions with the study nurse, who will encourage them to perform daily home evaluations at the week 1 and each of the 2-month visits.

The thermometry intervention will be the same to all study participants and has been adapted from previous experiences in thermometry use [6–8]. The intervention has been designed to enhance self-care through a thermometry device to be used by participants themselves or with help from their caregiver. Each patient will be instructed at the beginning of the study about foot care and how to perform the thermometry following a standardized procedure demonstrated on videos.

#### Assessment of intervention adherence and fidelity

We plan to perform a process evaluation to evaluate fidelity of the intervention. The objective of this evaluation will be to: (1) determine if participants know how to use the TempStat™, (2) determine if participants receive SMS and voice messages, (3) determine if participants understand the messages, and (4) obtain opinions from the participants about feedback received from the nurse when they call because of an alarm sign. In addition, we will interview study nurses to collect their impressions about difficulties and limitations during the study period [27].

### Periodic assessments

Participants will be encouraged to maintain regular visits with their treating physician in the outpatient clinic. Each hospital will follow their standard of care for diabetes management without restrictions. Also participants will be asked to visit the diabetes clinic every 2 months for a general checkup and lower extremity evaluation by a nurse evaluator. If the nurse identifies an ulcer or any diabetic complication in a patient, the patient will receive conventional hospital treatment. At each 2-month visit, participants will: (1) complete a questionnaire about diabetes treatment, caregiver presence, and use of insoles and/or orthopedic shoes, and (2) have their weight and blood pressure measured. Glycated hemoglobin (HbA1c) will be measured at 6 and 12 months. In addition, information from participants’ temperature-recording logbooks will be recorded.

### Outcome measures

#### Primary outcome measure

The primary outcome is foot ulceration occurring at any point during the 12-month study period after randomization. Using the American Diabetes Association criteria [28, 29], foot ulceration will be defined as any break in the cutaneous barrier that usually extends through the full thickness of the dermis. The evaluator, blind to the intervention allocation, will be a trained nurse.

There will be two ways of identifying if a patient has developed foot ulceration: (1) during the bimonthly clinical nurse evaluations, and (2) if an alarm sign has been noted and prompted the participant to seek clinical evaluation (see Table 1).

**Table 1:** Primary and secondary outcomes

Outcomes	Source of data	
	measure	End-point
Ulceration	Clinical evaluation by a trained nurse Participant’s report	Every 2 months until 12 months Any time during the 12 months
Adherence to daily temperature measurement	Logbook	Every 2 months until 12 months
Report of an alarm sign to the nurse	Nurse report	Every 2 months until 12 months
Report of an alarm sign in the logbook	Logbook	Every 2 months until 12 months
Dose-response analysis of SMS and voice messages	Automated system	Every 2 months until 12 months
Glycated hemoglobin	Blood sample	At 6 and 12 months
Sub-group analyses, in all participants		
Caregiving status	Participant’s report	Every 2 months until 12 months
Use of insoles and/or orthopedic shoes	Participant’s report	Every 2 months until 12 months
Sub-group analyses, intervention arm only		
Type of recipient (patient versus caregivers)	Participant’s report	Every 2 months until 12 months

#### Secondary outcome measures

The following have been pre-defined as secondary outcomes:

1. Adherence to daily temperature measurement: based on patient self-report of foot temperature monitoring through use of daily logbook
2. Report of an alarm sign to the nurse: frequency of alarms signs reported to the study nurse between study arms will be compared
3. Report of an alarm sign in the temperature-recording logbook: frequency of alarms signs reported in the patient’s logbooks between study arms will be compared
4. Dose-response analysis of SMS and voice messages: per protocol analysis
5. Glycated hemoglobin control targets: reduction of 1 % or more of glycated hemoglobin after 1 year of follow-up

#### Pre-specified sub-group analyses

In all participants, by (1) caregiving status, and (2) use of insoles and/or orthopedic shoes.

In the intervention arm only, by type of recipient (patient versus caregivers) of the messaging intervention.

### Sample size

Based on previous randomized trials and reports of ulceration in high-risk patients, we expect that 8.5 % of subjects in the thermometer plus SMS treatment group will develop ulcers during the evaluation period and that 30 % of subjects in the thermometer-only group will develop foot ulcers [6, 7]. With a power of 0.9 and an alpha of 0.05, we require a sample size of 78 subjects in each group to find an absolute change of 21 %, a reduction from 30 % to 8.5 %. Anticipating a 10 % dropout rate, we plan to enroll 86 subjects in each study group.

### Statistical analysis

The intention-to-treat principle will be performed comparing both study arms. To compare rates of ulceration between arms, we will perform an unadjusted logistic regression, specifically:

$$ \mathrm{logit}\ \left(\mathrm{P}\left(\mathrm{ulcer}\right)\right) \approx \upbeta 0 + \upbeta 1.\mathrm{intervention}+\upbeta 2.\mathrm{site} $$

where a logit function of the probability (P) of having ulcer depends on β0, the constant coefficient; β1 that indicates the intervention (1 if the participant is in the intervention arm, and 0 if otherwise), and β2 that refers to the coefficient of the study site (hospital).

Ulceration is the binary outcome and the intervention is the mixed modality application of thermometer use plus mHealth (SMS and voice messages). These analyses will include all retained participants, regardless of the number of visits attended. A finding that the coefficient is significantly greater than zero signifies that the mHealth intervention strategy has a significantly positive impact on the reduction of diabetic foot ulceration rates.

Evaluation of other outcomes will be performed according to a pre-defined approach.

#### Sub-group analysis

We will assess treatment effects for a specific patient characteristic: (1) caregiving status, assistance provided to the patient with (a) basic activities of daily living, or (b) in the identification, prevention, or treatment of diabetes or any disability, and (2) use of insoles and/or orthopedic shoes.

Additional sub-group analyses, in the intervention arm only: exploration of the primary outcome by the type of recipient (patient versus caregivers) of the messaging.

### Ethical issues

An essential element of this randomized trial, mainly related to the ethics of the study [30], was the decision to provide thermometry to all study participants. The researchers considered that the evidence available on the efficacy of thermometry interventions for the prevention of foot ulcers [6–8] was sufficient to incorporate it as part of the standard of care. This decision was made despite the fact that such practice is not currently available in the study setting or in many LMICs, thus favoring potential benefits rather than harms. Designing a randomized trial where the control group would not receive a thermometry device would be accompanied by a higher incidence of DFU in this group, a fact that was deemed not appropriate or ethical. Therefore, the decision was to standardize the provision of the thermometry device to all study participants where the only difference between study arms was the mHealth component, SMS and voice reminders, assigned only to one of the study arms.

The study protocol, informed consent templates, and questionnaires have been reviewed and approved by the Institutional Review Board at Universidad Peruana Cayetano Heredia in Lima, Peru. In addition, participating hospitals in the study will receive the protocol and consents for approval.

#### Monitoring, quality control and data management

Standard policies of the Universidad Peruana Cayetano Heredia for the development and review of the protocol will be followed, as well as policies related to adherence, safety procedures and information management. The Trial Steering Committee will be composed of the study coordinator, co-investigators, principal investigators and the Institutional Review Board of the Universidad Peruana Cayetano Heredia, who will provide trial oversight.

According to our Data Monitoring Plan, we will perform quality control at multiple stages, which include: (1) the use of manuals for data collection, (2) weekly meetings with study nurses, (3) updates training about protocol procedures, (4) duplicate data entry to the database, and (5) the ongoing review of the descriptive statistics of the trial data by the principal investigators with quality control review of selected data, looking for inconsistency, missing data and outliers. The databases will be encrypted and password-protected to ensure confidentiality. Close cooperation between the study coordinator, the data manager, and other members of the study team will be established to allow the tracking of the progress of the study to solve problems that arise during implementation and to address other issues on time.

With regards to data monitoring, given the pragmatic nature of the intervention, we will not establish an independent Data Safety and Monitoring Board (DSMB), as guidance indicates that a “DSMB is specifically required for multi-site clinical trials with interventions that entail risk(s) to participants” [31]. Our study is a phase III pragmatic clinical trial with a positive balance between harm-benefits for all the study participants. Data manager and investigators will be responsible for procedures of data monitoring.

## Discussion

This study makes two principal contributions towards the prevention of DFUs. First, the introduction of using messages to promote self-management of foot care in diabetes as well as using reminders as a strategy to improve adherence to daily home-based measurements. Secondly, the implementation of a thermometry-based strategy complemented by SMS and voice messages in an LMIC setting, with wider implications for scalability.

A recent systemic review found that, to date, a few studies with high-quality evidence show that DFUs can be prevented by complex interventions. Our proposal uses thermometry to reduce the incidence of foot ulcers in high-risk diabetic patients, which is an innovative and cost-effective preventative approach that promotes self-care at home to empower patient health-management with recognition of local cultural and economic limitations to healthcare access. Despite its promising expectation, patient compliance to at-home monitoring is an expected area of concern.

To address these concerns, we propose concurrent use of SMS and voice messages to remind patients to perform thermometry and then to assess how messages impact the progression to foot ulceration. SMS, an mHealth tool, has been shown to have better health outcomes for various diseases. A recent systematic review found that SMS reminders were useful in several clinical applications: adherence to antiretroviral and tuberculosis medications, and smoking cessation [32]. Data from Peru show some patients with chronic disease had difficulties reading SMS [33], thus we decided to additionally use voice messages to evaluate the impact of these communication strategies in prevention of ulceration of the diabetic foot.

Thermometry and SMS technologies have both been shown to have enormous potential in high-income countries, thus we propose to evaluate their utility in Peru, while combining the modalities to further enhance their impact. Our proposed methodology creates an early warning system for DFUs using few healthcare resources, which is a critical attribute for success in LMIC settings. We take advantage of the relatively high penetration of mobile phone technology – SMS are free for receivers, i.e., study participants – and our ability to deliver a low-cost thermometry device directly to our most at-risk patients. These tools have the potential to replace conventional diabetic care approaches in LMICs and to provide wide coverage in both urban and rural areas. We strongly believe that interventions such as the one proposed in this study may be able to extend “ulcer-free days” in patients in diabetic foot remission [34–36].

This project is being conducted as part of the The Global Alliance for Chronic Diseases (GACD) Diabetes Program, www.gacd.org. The GACD brings together the world’s largest funders of medical and health research to fund research on non-communicable diseases, with a collective investment of over US$100 million, across the vast and diverse GACD Research Network. The GACD Diabetes Program is the second of three research initiatives, with over US$26 million in funding across 17 projects in 22 countries. Each research project is conducted through a unique partnership between investigators from institutions in high-income countries as well as LIMCs. The aim is to build a collaborative group of international researchers who meet annually and participate in joint working groups. All research teams actively participate in collaborations on implementation science, common to the studies, with the ultimate goal of translating evidence into policy.

We believe the creativity of this protocol offers the potential to tackle a difficult problem from a unique angle and, therefore, could have a substantial impact on DFU prevention not only in Peru but also in much of the world.

### Trial status

At the time of submission, this trial is in the process of participant recruitment.

## Additional files

Additional file 1: Message design and evaluation. (DOCX 12 kb) Additional file 2: mHealth component. (DOCX 11 kb)

## Abbreviations

DFN: diabetic foot neuropathy
DFU: diabetic foot ulcer
DSMB: Data and Safety Monitoring Board
GACD: Global Alliance for Chronic Diseases
HbA1c: glycated hemoglobin
IWGDF: International Working Group on the Diabetic Foot
LMIC: low- and middle-income country
SMS: Short Message Service
SWF: Semmes-Weinstein monofilament
T2DM: type 2 diabetes mellitus
VPT: vibration perception threshold
