# Supplementary material for: Implementation of foot thermometry plus mHealth to prevent diabetic foot ulcers: study protocol for a randomized controlled trial
Source: Trials. 2016 Apr 19;17:206. doi: 10.1186/s13063-016-1333-1 (PMC4837616; doi:10.1186/s13063-016-1333-1)
Supplement: Additional file 1: — Message design and evaluation. (DOCX 12 kb) [file 13063_2016_1333_MOESM1_ESM.docx]

## Additional file 1. Message design and evaluation

The content of the SMS and phone messages will be designed using previous team experience and theory from literature review. Two kinds of messages will be provided to intervention arm participants: i) Reminder messages to promote temperature measurement, and ii) Messages to promote foot care.

The content-based structure of the messages will include:

- Positive consequences (prevent ulceration, foot care) and How to measure foot temperature using the TempStat™.
- Negative consequences (ulceration) and How to avoid walking barefoot.
- Potential barriers (it is difficult to you) and How to ask for help/support from family/friends.

These messages will be validated between the research team prior to field-site validation with 10 individuals with diabetes to assess patients’ comprehension of the messages.
