# Supplementary material for: Implementation of foot thermometry plus mHealth to prevent diabetic foot ulcers: study protocol for a randomized controlled trial
Source: Trials. 2016 Apr 19;17:206. doi: 10.1186/s13063-016-1333-1 (PMC4837616; doi:10.1186/s13063-016-1333-1)
Supplement: Additional file 2: — mHealth component. (DOCX 11 kb) [file 13063_2016_1333_MOESM2_ESM.docx]

## Additional file 2. mHealth component

Engineers at Universidad Nacional de Ingenieria in Lima, Peru have developed the mHealth system, which automatically sends SMS and voice messaging. The system has to record information about participants, for example, how many SMS and voice messages the system sent, how many calls are answers, and how long last the call.
